# A Micromanipulator and Transporter Based on Vibrating Bubbles in an Open Chip Environment

**DOI:** 10.3390/mi8040130

**Published:** 2017-04-18

**Authors:** Liguo Dai, Niandong Jiao, Xiaodong Wang, Lianqing Liu

**Affiliations:** 1 State Key Laboratory of Robotics, Shenyang Institute of Automation, Chinese Academy of Sciences, Shenyang 10016, China; dailiguo@sia.cn (L.D.); wangxiaodong@sia.cn (X.W.); 2 University of Chinese Academy of Sciences, Beijing 100049, China

**Keywords:** vibrating bubble, micromanipulation, cell trapping, open chip environment

## Abstract

A novel micromanipulation technique of multi-objectives based on vibrating bubbles in an open chip environment is described in this paper. Bubbles were created in an aqueous medium by the thermal energy converted from a laser. When the piezoelectric stack fixed under the chip vibrated the bubbles, micro-objects (microparticles, cells, etc.) rapidly moved towards the bubbles. Results from numerical simulation demonstrate that convective flow around the bubbles can provide forces to capture objects. Since bubbles can be generated at arbitrary destinations in the open chip environment, they can act as both micromanipulators and transporters. As a result, micro- and bio-objects could be collected and transported effectively as masses in the open chip environment. This makes it possible for scientific instruments, such as atomic force microscopy (AFM) and scanning ion conductive microscopy (SICM), to operate the micro-objects directly in an open chip environment.

## 1. Introduction 

The manipulation of individual micro-objects, which usually includes capture, transport, rotation, and isolation, is becoming a critical technology for micro-assembly and biomedical applications. At the micro-scale level, particles and cells can be moved and patterned by traditional methods, such as optical traps [1,2,3,4], dielectrophoresis forces [5,6,7], acoustic waves [8,9,10,11], and magnetic fields [12,13,14,15]. Moreover, many new techniques and tools have drawn more attention because of their great potential in lab on a chip applications, one of which being microbubbles. Many researchers have focused on the novel and special function of bubbles in microfluidic systems, such as pumps [16,17], actuators [18,19], mixers [20,21], and valves [22,23], as well as in other devices [24,25,26,27].

Bubbles have become a versatile tool for various lab on a chip and micromanipulation applications, and many innovative devices have been explored in recent years [28]. Microbubbles can be used as focusing agents when the acoustic streaming flow exerts a large enough shear force on vesicles in the flow, so it is possible to be developed for cell manipulation, cell-wall permeation and microfluidic devices [29]. Further, oscillating bubbles are capable of size- and density-based selective trapping of particles [30]. With other on-chip manipulation methods, both millimeter- and micro-sized objects could be captured, carried, and released by oscillating mobile bubbles [31]. Particle trapping and manipulation can also be completed by optothermally-generated bubbles, rather than oscillating bubbles [32]. Bubbles used for manipulation are generally controlled by the electrowetting-on-dielectric (EWOD) [31,33,34,35], or optical methods [32,36,37,38,39]. In an aqueous medium, bubbles transported by AC-electrowetting, and oscillated by piezo-actuator, are capable of capturing, carrying, and releasing objects [31,33]. Using two arrays of EWOD electrodes, twin bubbles driven and transported simultaneously can attract and capture beads and fish eggs [34,35]. Because bubbles are actuated by alternating the EWOD chip, the trajectory of bubbles and micro-objects is limited by design of the chip. Thus, another mechanism of generation and control of bubbles is proposed. In fluidic oil chambers, micro bubbles act as microrobots for manipulation and assembly, which are controlled by optically-induced heating and thermocapillary effects [36]. Micro-object trapping and manipulation can be completed by an optothermal bubble, due to the convective flow, surface tension, and pressure forces [32]. When a bubble is generated at the top of a gold film, a convective flow was formed around the bubble, so that a particle can be moved towards the bubble by the convective flow induced by a temperature gradient. After the particle approaches the bubble, it is trapped on the bubble’s surface, because of the balance of pressure force and surface tension force along the radial direction of the bubble. Micro-objects can be carried along with the optically-controlled bubble to any desired location, while the closed fluidic chamber has limited the cooperation with other scientific instruments and technology. In addition, manipulation of the micro-particles and cells can also be completed with a non-contact method, where disk-shaped hydrogel microrobots actuated by laser-induced cavitation bubbles are used to draw patterns of cells and microgels [37]. Since the optothermally-induced fluid flow can trap and transport bio-objects, a micromanipulation platform based on bubbles is capable of manipulation and patterning [38]. However, since each microrobot or manipulator can only pattern and manipulate a single cell at a time, efficiency remains to be improved.

Here we present a novel manipulation method based on vibrating bubbles in an open chip environment, by which micro-objects (microparticles, cells, etc.) could be collected and transported efficiently as mass in the open chip environment. Both optothermally-generated bubbles [40] and oscillating bubbles [41] are popularly used in lab on a chip community, and have been well-studied for micromanipulator applications. However, using both techniques in one device is proposed for the first time in this paper. Compared with the methods of micro-objects manipulation mentioned above, this new technique can collect and move particles, as well as cells, in an open micro chamber without top glass. This technology is expected to function as a transporter for particle manipulation and transportation, collaboratively used in unclosed operating environments of other scientific equipment (atomic force microscopy (AFM) and scanning ion conductive microscopy (SICM), etc.).

## 2. Materials and Methods

### 2.1. Materials

In our experiment, the chip consisted of polydimethylsiloxane (PDMS, SYLGARD184, Dow Corning Holding Co., Ltd., Midland, MI, USA), glass substrate and gold layer, and the pre-polymer of PDMS was a mixture of base and curing agent. Purified deionized water was filled in the reservoir of chip when the manipulation objects were microballs. The balls with diameter of 50 to 100 μm were made of barium titanate glass (BTG). In the experiment of manipulating cells, Human Embryonic Kidney (HEK) 293 cells and pandorina morum cells acted as micro-objects. Experimental strains of HEK 293 cells were obtained from China Center for Type Culture Collection (Wuhan, China), and the strains of *pandorina morum* were provided by the Freshwater Algae Culture Collection at the Institute of Hydrobiology (Wuhan, China). The fluid in the reservoir was replaced by culture medium (Eagle’s Minimum Essential Medium and Tris-acetate-phosphate medium respectively) in the experiments of manipulating cells with oscillating bubbles. The diameter of HEK 293 cells was about 20 μm, while the diameter of morum cells was 30 μm.

### 2.2. Methods

**Figure 1:**
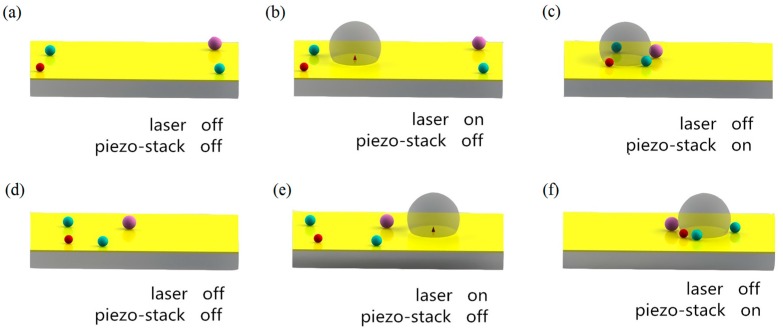
Collecting and transporting of micro-objects by oscillating vibrating bubbles: (**a**) micro-objects distributed on the chip; (**b**) a bubble generates on the chip; (**c**) piezoelectric stack is turned on and the particles are collected by the oscillated bubble; (**d**) the bubble bursts and the micro-objects disperse; (**e**) another bubble appears on the chip; (**f**) the new bubble collects these objects again.

The new method can manipulate and trap multi-objects and cells at an arbitrary destination from relatively long distances away on the chip, and then transport them to a new location by another optothermally-generated bubble. As shown in Figure 1, to manipulate the micro-objects, a bubble is created on a chip coated with a gold layer. The diameter of the bubble is related to the intensity and irradiation time of the laser, for the bubble is produced by optically-induced heating. When the bubble is vibrated by a piezoelectric stack, objects were attracted to the bubble by convective flow. Using a high-voltage signal, the working distance of the micro bubbles may reach the millimeter scale. Theoretical analysis and simulations were conducted in our studies that reveal that micro-objects are driven towards the bubble vibrated by the piezo-actuator by heat-induced convective flow. If we turned on the laser, the bubble would increase in size continuously and explode, causing the micro-objects collected previously to disperse. When the frequency of the wave applied to the piezo-stack was transformed to the bubble’s resonance frequency, the bubble could also be damaged. By changing the position of the next bubble after the previous one has burst, the dispersed micro-objects could be re-collected and moved to the new destination. Further, the moving distance of the particles could be as long as the channel in the chip. Simultaneous manipulation and transportation of multitarget objectors could be completed in an unclosed chip. 

### 2.3. Experiment Setup

**Figure 2:**
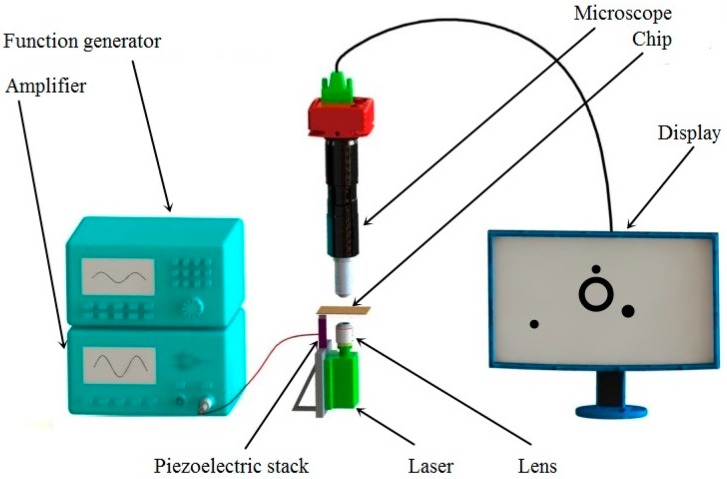
Schematic of the system setup.

The experiment system is shown in Figure 2, where a semiconductor laser (405 nm wavelength, 0–400 mW power), and a lens (25X, NA = 0.40), were used to provide sufficient power for the generation of a bubble. The laser and lens were fixed to a manual stage so that the position of the bubble generated was controllable and variable. A piezoelectric stack (PK2FMP2, Thorlabs Inc., Newton, NJ, USA), driven by an arbitrary waveform generator (ArbStudio 1102, Teledyne LeCroy Inc., Chestnut Ridge, NY, USA), together with an amplifier (33502A, Keysight Technologies Inc., Palo Alto, CA, USA), vibrated the micro bubble on the chip. The drive voltage of the piezo-actuator range was 0–75 V, and the displacement at 75 V was 11.2 μm. The chip was made up of a 1.2-mm-thick slide glass, and a small PDMS reservoir. A 50 nm thin-film layer gold layer was sputtered on the glass to absorb and transfer the energy of the laser beam. Other devices in this system included an optical microscope (1-60191D, Navitar Inc., Rochester, NY, USA), a camera (FL2G-13S2, Point Grey Research Inc., Richmond, BC, Canada), a computer, and a long pass filter (FELH0450, Throlabs, Newton, NJ, USA) with a 450 nm cut-on wavelength, which can reject the laser light into the microscope.

### 2.4. Fabrication of Chip 

**Figure 3:**
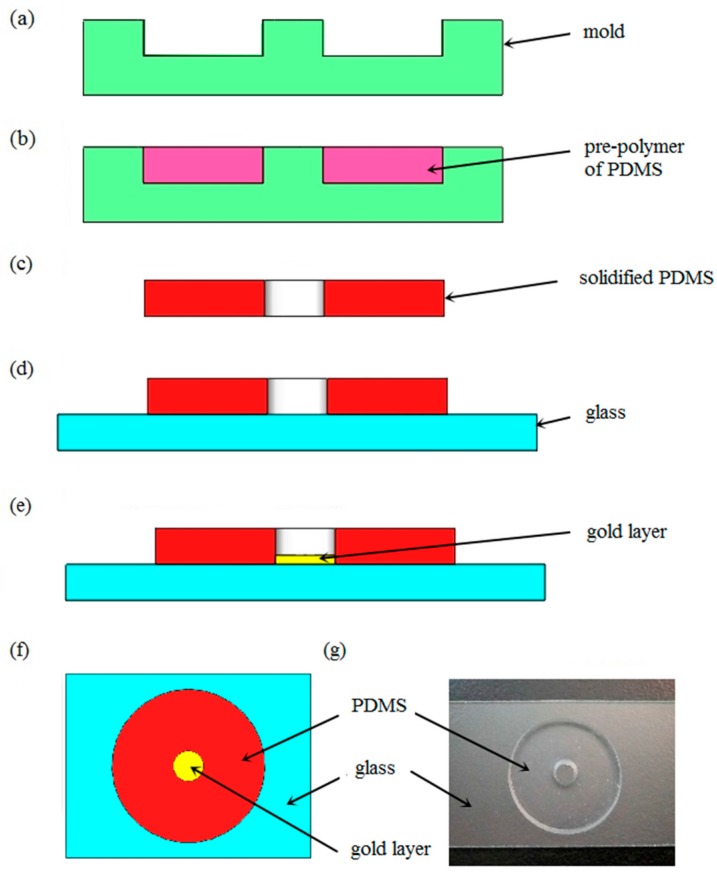
(**a**–**e**) Scheme of the fabrication process of the chip: (**a**) fabricating a mold; (**b**) pouring liquid pre-polymer into the pattern die and heating; (**c**) removing the cured polydimethylsiloxane (PDMS) copy; (**d**) combining the PDMS with a slide glass; (**e**) gold layer is sputtered on the glass; (**f**) schematic top view of the chip; (**g**) actual picture of the chip without gold layer.

The microfluidic chip, consisting of a glass substrate, fluid reservoir, and gold layer, has a simple design and can be fabricated rapidly. The reservoir was manufactured with PDMS, an elastomeric material [42,43]. Because of its physical and chemical properties, such as transparency, insulation, and nontoxicity, PDMS has become one of the most actively developed polymers for microfluidics. In contrast to general microfluidic chips, the chips used in these experiments were unclosed. The manufacturing process can be divided into five steps, as illustrated in Figure 3a–e. First, an acrylic mold is designed in a computer-aided design program and produced with machine tools. A pre-polymer of PDMS in the liquid state is then injected into the mold and cures gradually at a temperature of 75 °C. In our experiments, the PDMS included two ingredients—a base and a curing agent. An elastomeric and cross-linked solid was generated when the vinyl groups of the base reacted with the silicon hydride groups of the curing agent. These two kinds of solution were mixed in a mass ratio of 10:1 to produce a replica. Approximately four hours later, the liquid pre-polymer solidified and conformed to the shape of the master. The solidified PDMS cast was then peeled away from the die. Following this, the PDMS structure was oxidized for five minutes and sealed tightly and irreversibly to the slide glass. Silanol groups formed on the surface of the PDMS by the oxidation of methyl groups so that it could seal to a range of materials other than itself, including glass, silicon, and polyethylene. Since the gold layer prevented the linkage of PDMS and glass, the last procedure is the sputtering of gold on the chip. The diameter and depth of the reservoir on the fabricated chip, shown in Figure 3f, is 3 mm and 2 mm, respectively.

## 3. Results and Discussion

### 3.1. Simulation

**Figure 4:**
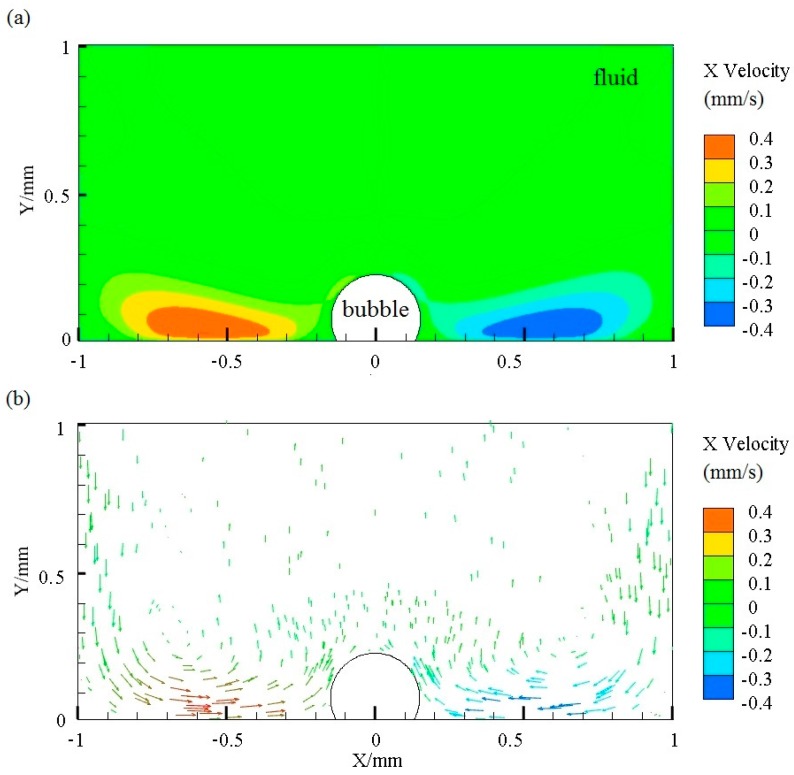
(**a**) Contours of X-velocity of the flow; (**b**) vectors of the flow. The simulation is conducted by ANSYS Fluent software. The diameter of the bubble is 236 μm, the size of the liquid zone is 2 mm × 1 mm, and the frequency and displacement of vibration is 10 kHz and 5 μm, respectively.

To provide theoretical guidance for the object trapping and manipulation process, computational fluid dynamics simulations were conducted using ANSYS Fluent software (Version 14.0, Pittsburgh, PA, USA). The goal of the simulations was to reveal the convective flow pattern around the oscillated bubble when the piezo-actuator was on. Micro-objects and cells could be moved by the force of the fluidic streaming, which is studied by experiments and numerical analysis. According to Navier-Stokes Equations [29,44], the motion of a viscous incompressible fluid can be described as:

(1) $$\frac{\partial \rho }{\partial t}+\nabla \left(\rho v\right)=0$$

where ρ is the mass density, t is time, and v is the fluid density. In the two-equation turbulence model [45,46,47], the eddy viscosity is defined by:

(2) $$\mu_{T}=\rho k/\omega$$

where $\mu_{T}$ is the velocity vector, *k* is the turbulence kinetic energy, and ω is the specific dissipation rate. To fulfill the simulation, a simplified two-dimensional model is used in our calculation. The size of the liquid zone was 2 mm × 1 mm, while the frequency of vibration was set as 10 kHz and the displacement of vibration was 5 μm. We employed a microscope (KH-7700, Hirox Inc., Tokyo, Japan) to obtain the radius of the bubble, and the height difference between the center of bubble and the interface of the chip. For a bubble with a radius of 118 μm, the height of the center from the chip was 58 μm, which indicates that the bubble is part spherical. The distribution of the X-velocity of flow can be obtained from the simulation results, as shown in Figure 4a. The maximum absolute value of velocity is approximately 400 μm/s. However, at the zone adjacent to the bubble, the rate of flow was relatively low and approaches zero, which correlates well with the experimental results. Figure 4b indicates that in the bottom area of the liquid, the direction of convective flow was towards the bubble, such that micro-objects and cells could be attracted by the bubble.

### 3.2. Generation of Bubble 

**Figure 5:**
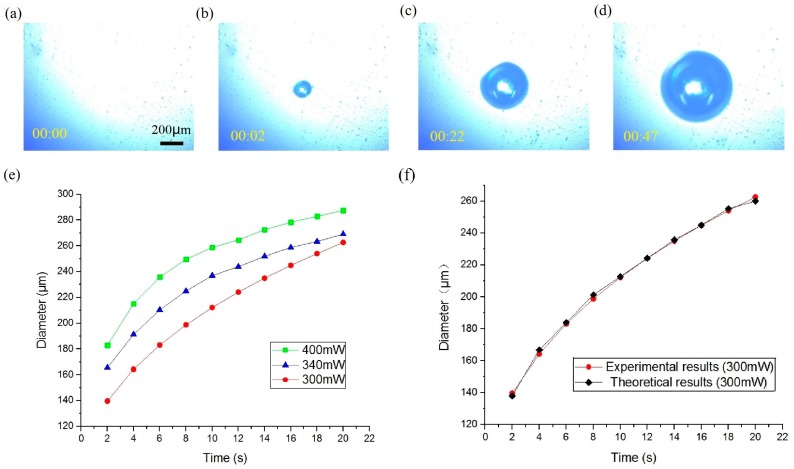
(**a**–**d**) Generation and expansion of bubble, the time stamp format is minute:second; (**e**) growth process of bubble under different conditions where the power of the laser differs; (**f**) comparison between experimental data and theoretical analysis results, where power is 300 mW.

The process of the generation and expansion of the microbubble is shown in Figure 5a–d. When the laser was focused on the liquid-solid interface by the lens, the gold layer absorbed the energy of the light and transferred it into thermal power, and thus the temperature of the liquid near the spot rose. An opto-thermal bubble then generated and expanded continuously because the solubility of the gas generally decreased with increased water temperature. In the initial 5 s, the diameter of the bubble rose quickly. However, the increasing speed gradually then reduced. The diameter of the bubble was determined predominantly by the irradiation time and the power of the laser, as shown in Figure 5e. The greater the power transferred into thermal energy, the more gas separated from the water. Thus, the volume of the bubble is in proportion to the quantity of heat generated. The radius of the bubble is related to the working time and intensity of the laser beam. The growth process of gas in solution can be described by the Lifshitz-Slyozov-Wagner theory [48]:

(3) $$V-V_{0}=kIt$$

where V is the volume of the bubble, $V_{0}$ is the initial volume of the bubble, I is the power of the laser, k is the efficiency of energy conversion, and t is the irradiation time. The volume of a part-spherical bubble is proportional to the third power of the radius (r) of the bubble:

(4) $$r^{3}=r_{0}^{3}+c^{-1}kIt$$

where $r_{0}$ is the initial radius of the bubble, and c is a constant representing the ratio of the volume and radius. As shown in Figure 5f, the experimental results agree to the theoretical curve strongly.

### 3.3. Manipulation of Microparticles

**Figure 6:**
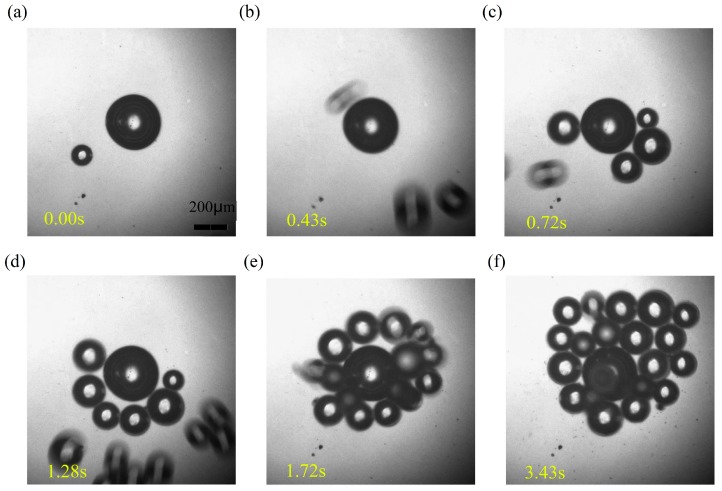
Collection process of microparticles by a bubble; the time format unit is seconds. The frequency and voltage of piezo-actuator is 8 kHz and 30 V; the diameter of bubble and micro glass balls is about 150 μm and 50–100 μm. (**a**) 0.00 s, (**b**) 0.43 s, (**c**) 0.72 s, (**d**) 1.28 s, (**e**) 1.72 s and (**f**) 3.43 s.

To manipulate micro-objects in a liquid reservoir, the function generator and amplifier were turned on to output a sinusoidal voltage, so that the microbubble generated previously was piezo-actuated. The microstreaming around the bubble then attracted objects to the surrounding area progressively, such that micro-objects were collected by the oscillating bubble. Figure 6 demonstrates how the BTG microparticles were captured individually. The diameter of these microballs ranges from 50 to 100 μm. When the bubble is vibrated, a nearby object is trapped initially, and another two balls move towards the bubble at a later stage. Provided the micureobjects were captured, the motion stopped before the streaming became week, as demonstrated by the simulations. Simultaneously, more and more objects were pulled to the bubble continually. The microballs moved quickly, with this manipulation process taking only three seconds.

**Figure 7:**
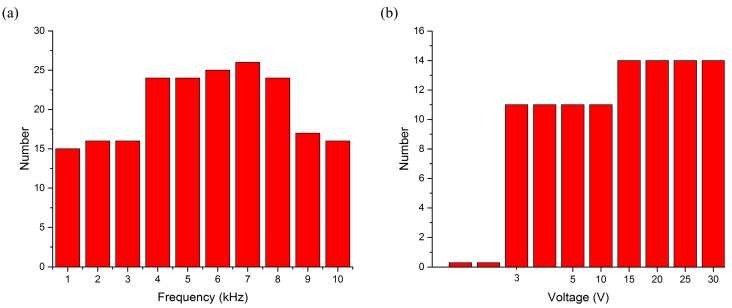
(**a**) Number of micro-objects collected by the bubble at different actuating frequencies; (**b**) number of the microballs collected by the bubble at different actuating voltages.

To demonstrate the collection ability of the vibrated bubble, the number of micro-objects trapped under different actuation conditions (for example, frequency and amplitude) was studied. Results are shown in Figure 7. In this experiment, there were 30 micro-objects in the liquid reservoir. When the signals were set at 30 V, a bubble could attract more microballs when the vibrating frequency was 4–8 kHz. When the frequency was lower than 500 Hz or higher than 15 kHz, no further objects could be collected. By maintaining a constant frequency of 10 kHz, and gradually increasing the waveform generation gradually, an increasing number of objects were trapped by the bubble. When the voltage was lower than 3 V, the glass balls did not move. Moreover, when the voltage was turned up to 15 V, the ability of the bubble reached its limit. Other conditions, such as the bubble diameter and waveform of the actuation signal, did not affect the number of collected objects.

### 3.4. Manipulation of Cells 

**Figure 8:**
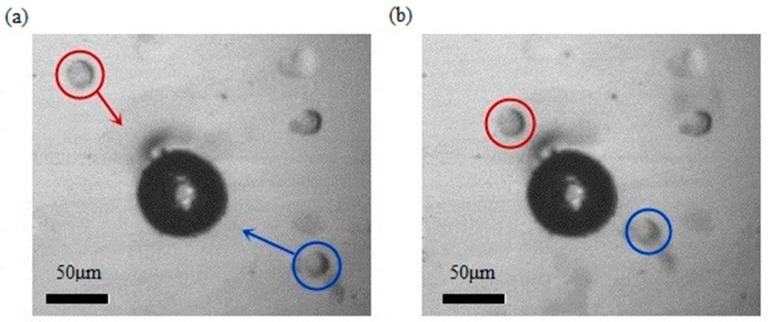
Moving and collecting process of HEK 293 cells by a bubble. (**a**) The cells marked with red and blue circles moves towards the bubble; (**b**) the two cells are collected by the oscillating bubble. The frequency and amplitude of control signal is 8 kHz and 30 V. The diameter of bubble is about 70 μm, and the diameter of the cells is 20 μm. This moving process takes only one second.

Besides glass balls, the bubbles could also be used to trap bio-objects, including cells. HEK 293 cells are often used in biological and medical experiments, since they are easy to transfer and culture. The process of collecting and manipulating HEK 293 cells using a piezo-actuated bubble is shown in Figure 8. To manipulate the HEK 293 cells, the opto-thermal bubble was generated in deionized water, as the high viscosity of the culture solution prevented the generation of bubbles. Culture solution and suspended HEK293 cells were then injected into the reservoir on the chip to replace water. HEK 293 cells were usually cultured adherently, and could be suspended in the medium. When the piezo-actuator was turned on, the cells moved towards the oscillated bubble. The diameter of bubble was 70 μm, while the diameter of HEK 293 cells was approximately 20 μm. The two cells in the picture were driven towards the bubble, with their moving distance being about 50 μm, and a speed of 50 μm/s. The frequency and voltage was set at 8 kHz and 30 V, respectively. In our experiments, the activity of the cells was not affected after collection.

**Figure 9:**
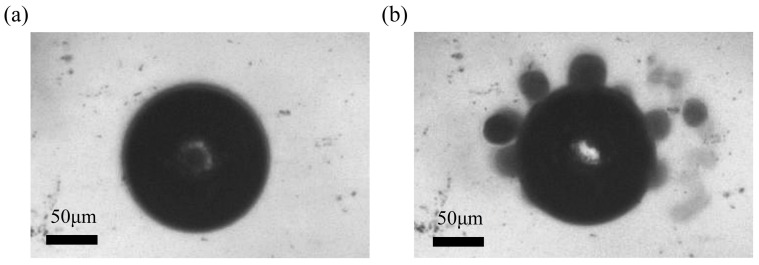
Collection of *pandorina morums* by an oscillated bubble. (**a**) No *morums* around the bubble when the piezo-actuator is off; (**b**) some *morums* are collected by the vibrating bubble. The frequency and amplitude of control signal is 8 kHz and 30 V. The diameter of bubble is about 130 μm, and the diameter of the cells is 30 μm. This collecting process takes five seconds.

In addition, the manipulation method can be used for trapping swimming *pandorina morum* cells. It is a promising application in biology, chemistry, physics, and medicine to manipulate cells with intrinsic motility, as the precise manipulation of the mobile microorganisms (such as bacterial and algal cells) remains difficult [49]. The feasibility and effectiveness of utilizing this technology to manipulate swimming *pandorina morum* cells was investigated, as shown in Figure 9. A bubble was generated on the chip using optically-induced heating. *Pandorina morums* then moved toward the oscillating bubble, and were trapped. Since cells with diameters of 30 μm were smaller than the bubble (whose diameter is more than 100 μm), some of them in the shade of bubble are invisible in the figures. Because the *morum* cells can be self-propelled, this manipulation process took about 5 s to collect these swimming cells far from the bubble, and the moving speed went up to 100 μm/s. The driven voltage used was 8 kHz and 30 V. In our experiments, the activity of *pandorina morum* cells was not affected, and the captured cells could swim away when the bubble was damaged. 

### 3.5. Transportation of Micro-Objects 

**Figure 10:**
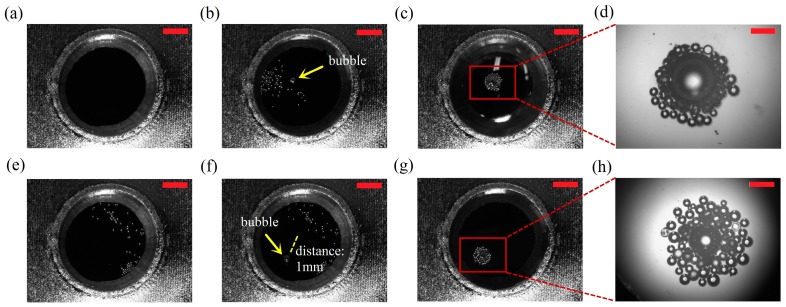
Collection and transportation of micro-objects: (**a**) liquid reservoir; (**b**) first bubble generates on the chip; (**c**,**d**) vibrated bubble attracts microballs—this manipulation takes about 3 s; (**e**) objects spread after the first bubble is damaged; (**f**) a new bubble appears at new location 1 mm far from the original position; (**g**,**h**) microballs are re-collected and transported by the oscillated bubble—the collection process takes 4 s. Scale bars in (**a**–**c**), (**e**–**g**) are 1 mm, and are 200 μm in (**d**,**h**).

In addition, we could collect micro-objects at different destinations by changing the position of the bubble. Because the mobile stage was connected with a laser and lens, we could generate bubbles at arbitrary locations, so that the transportation of microballs was complete. When a bubble burst or was damaged, the micro-objects could be re-collected at a different destination where a new bubble was generated. Figure 10 illustrates the procedures of the collection and transportation of the micro-objects. The diameter of bubble in Figure 10b–d is more than 300 μm, while the diameter of bubble in Figure 10f–h is about 200 μm, and the diameter of mciro balls is 50 to 100 μm. The moving distance of the balls was more than 2 mm in the chip. The frequency and amplitude of the input signal of the piezo-actuator was 8 kHz and 30 V, respectively. The first bubble was generated on the chip and the particles were captured. Since a bubble disappeared at its natural frequency, it could be damaged by changing the frequency of the piezoelectric stack, resulting in the collected objects redispersing in the aqueous media. In addition, if we turned on the laser again, the bubble grew continuously to its limit and broke. If we moved the laser spot to a new destination 1 mm away from the original location and the second bubble is created, the microballs collected around this new oscillatory bubble. This manipulation process was repeatable and micro-objects could be transported continuously. The working distance of the vibrated bubble could cover the whole chip. Thus, the microballs can be transported by the bubble over considerable distances.

## 4. Conclusions

A novel micromanipulation and transportation technology using opto-thermally generated and piezo-actuated bubbles is proposed in this paper. Although optothermal generation and acoustic oscillation of microbubbles have been well studied and applied to micromanipulator and lab-on-a-chip, using these techniques in one chip has not been realized so far. The ability of the bubble to capture objects, which is demonstrated by the computational fluid dynamics simulation, is related to characteristics of the control signal. Since the bubble can be generated at arbitrary locations in the open chip environment, the microballs and cells can be collected and transported efficiently as a mass. Manipulation in open chip environment, along with the function of collection and transportation of micro-objects, make it a promising micromanipulation method. The method based on vibrated bubbles is expected to cooperate with other scientific instruments, such as AFM and SICM, under the open operating condition. The advantages of this manipulation technique, such as versatility and simplicity, makes it a good candidate for actuation of self-sufficient, stand-alone microfluidic systems [50], if the laser and acoustic wave sources can be miniaturized further.
